# Interactions of *Streptococcus suis* serotype 2 with human meningeal cells and astrocytes

**DOI:** 10.1186/s13104-015-1581-2

**Published:** 2015-10-26

**Authors:** Jean-Philippe Auger, Myron Christodoulides, Mariela Segura, Jianguo Xu, Marcelo Gottschalk

**Affiliations:** Faculty of Veterinary Medicine, Research Group on Infectious Diseases of Swine (GREMIP) and Swine and Poultry Infectious Diseases Research Center (CRIPA), University of Montreal, 3200 Sicotte Street, Saint-Hyacinthe, QC J2S 2M2 Canada; Neisseria Research Group, Molecular Microbiology, Clinical and Experimental Sciences, University of Southampton Faculty of Medicine, Southampton, UK; Collaborative Innovation Center for Diagnosis and Treatment of Infectious Diseases, National Institute for Communicable Disease Control and Prevention, Chinese Center for Disease Control and Prevention, Beijing, China

**Keywords:** *Streptococcus suis* serotype 2, Pig pathogen, Zoonosis, Meningeal cells, Astrocytes, Meningitis

## Abstract

**Background:**

*Streptococcus suis* serotype 2 is an important porcine pathogen and emerging zoonotic agent responsible for meningitis, of which different sequence types predominate worldwide. Though bacterial meningitis is defined as an exacerbated inflammation of the meninges, the underlying astrocytes of the *glia limitans superficialis* may also be implicated. However, the interactions between this pathogen and human meningeal cells or astrocytes remain unknown. Furthermore, the roles of well-described virulence factors (capsular polysaccharide, suilysin and cell wall modifications) in these interactions have yet to be studied. Consequently, the interactions between *S. suis* serotype 2 and human meningeal cells or astrocytes were evaluated for the first time in order to better understand their involvement during meningitis in humans.

**Results:**

*Streptococcus suis* serotype 2 adhered to human meningeal cells and astrocytes; invasion of meningeal cells was rare however, whereas invasion of astrocytes was generally more frequent. Regardless of the interaction or cell type, differences were not observed between sequence types. Though the capsular polysaccharide modulated the adhesion to and invasion of meningeal cells and astrocytes, the suilysin and cell wall modifications only influenced astrocyte invasion. Surprising, *S. suis* serotype 2 induced little or no inflammatory response from both cell types, but this absence of inflammatory response was probably not due to *S. suis*-induced cell death.

**Conclusions:**

Though *S. suis* serotype 2 interacted with human meningeal cells and astrocytes, there was no correlation between sequence type and interaction. Consequently, the adhesion to and invasion of human meningeal cells and astrocytes are strain-specific characteristics. As such, the meningeal cells of the leptomeninges and the astrocytes of the *glia limitans superficialis* may not be directly implicated in the inflammatory response observed during meningitis in humans.

## Background

*Streptococcus suis* is an important porcine bacterial pathogen and emerging zoonotic agent responsible for sepsis and meningitis [1]. Of the thirty-five described serotypes, based on the presence of the capsular polysaccharide (CPS) or its respective genes, serotype 2 is regarded as the most widespread and virulent, responsible for the majority of porcine and human cases of infection worldwide [2]. In fact, 97 % of the approximately 1300 serotyped *S. suis* cases of infection in humans were caused by the serotype 2, of which nearly 70 % presented clinical signs of meningitis [2]. Furthermore, this pathogen is the first cause of adult bacterial meningitis in Vietnam, the second most common in Thailand, and the third most frequent cause of community-acquired bacterial meningitis in Hong Kong [2]. The use of multilocus sequence typing has identified four predominate serotype 2 sequence types (ST): the ST1 in Eurasia, the ST7, responsible for the human outbreaks of 1998 and 2005, in China, and the ST25 and ST28 in North America [2]. In addition, porcine and human cases of infection have been attributed to ST25 and ST28 strains in Thailand and Japan, though less frequently [2]. Moreover, differential interactions have been observed between STs using various cell types from different species [3, 4].

Of the multitude of *S. suis* serotype 2 virulence factors described [1], the CPS, suilysin (SLY) and cell wall modifications have been well-characterized. The CPS is a critical factor implicated in a multitude of functions, most importantly in the resistance to phagocytosis [5, 6]. Meanwhile, the SLY is a hemolysin responsible for causing cell cytotoxicity [7]. Finally, cell wall modifications, such as the D-alanylation of the lipoteichoic acid (LTA) and N-deacetylation of the peptidoglycan (PGN), are known to interfere with the immune response; the D-alanylation of the LTA was also shown to modulate adhesion to and invasion of endothelial cells [6, 8, 9].

The interactions between *S. suis* serotype 2 and certain cells of the central nervous system (CNS) have been studied, including porcine brain microvascular endothelial cells (pBMEC) and porcine choroid plexus epithelial cells (CPEC), as well as murine microglia and astrocytes [4, 10–12]. However, few studies have used human cells: only the interactions with human BMEC (hBMEC) and choroid plexus papilloma cells have been reported [13, 14], while those with meningeal cells and other cells of the human CNS remain unknown. The meninges are composed of the dura mater and leptomeninges: the latter are formed of the pia mater and arachnoid mater together with the trabeculae that traverse the cerebrospinal fluid (CSF)-filled subarachnoid space (SAS) [15]. The pia mater overlies the *glia limitans superficialis*, a layer of compact astrocytes that surrounds the brain and forms a barrier [16]. While astrocytes play a crucial role in cerebral homeostasis, they also participate in inflammation, though this role has only begun to be studied [17, 18]. Consequently, astrocytes possess a multitude of pattern recognition receptors involved in the innate immune response, including Toll-like receptors (TLRs) and nucleotide-binding oligomerization domain (NOD)-like receptors (NLRs) [18]. As such, these cells are a source of certain inflammatory mediators [18].

Penetration of the CPEC that form the blood-CSF barrier by *S. suis*, from the underlying blood vessels, is a proposed portal of entry into the CNS [14, 19]. Consequently, the leptomeninges and underlying astrocytes of the *glia limitans* are possibly implicated in both porcine and human *S. suis* infections, which are characterized by meningitis (inflammation of the leptomeninges) often accompanied by inflammation of the surrounding CNS tissues, such as the *glia limitans* [20]. The interactions of different important meningitis-causing human bacterial pathogens with these cells have already been studied [21–24].

In this study, the interactions between different *S. suis* serotype 2 strains and human meningeal cells or astrocytes, including certain well-characterized virulence factors, were evaluated for the first time in order to better understand the implication of the meninges and underlying CNS tissue during meningitis in humans.

## Results and discussion

### *Streptococcus suis* serotype 2 adheres to but rarely invades human meningeal cells and these interactions are modulated by the CPS

**Fig. 1:**
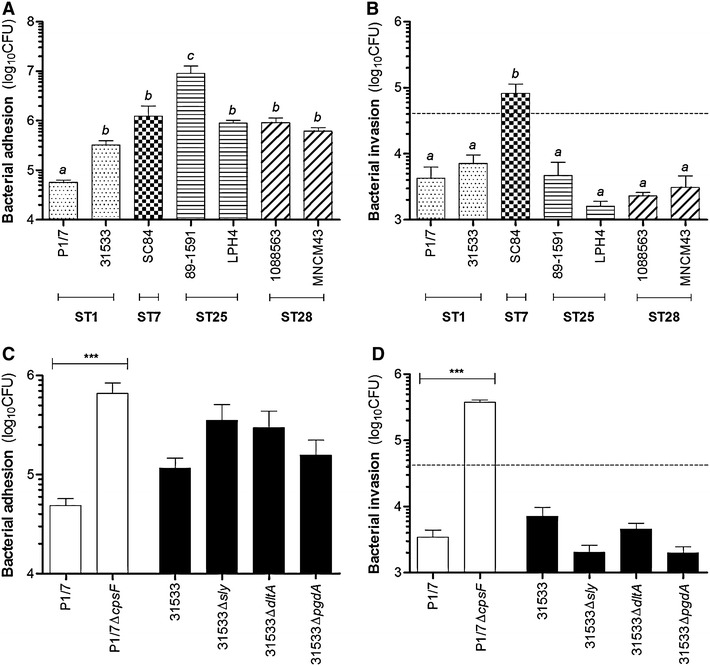
Interactions of *S. suis* serotype 2 with human meningeal cells. Bacterial adhesion (**A**) and invasion (**B**) of human meningeal cells by the *S. suis* strains belonging to different sequence types (STs), and adhesion (**C**) and invasion (**D**) of human meningeal cells by the mutant strains. Results are expressed as log_10_ mean ± SEM CFU obtained from three independent experiments. *Dotted lines* indicate a ratio of 1 intracellular bacterium/cell. The use of different letters (*a*, *b* or *c*) indicates a significant difference between groups (p < 0.01); ***(p < 0.001)

Though a high blood bacterial load is often regarded as crucial for the development of meningitis by extracellular bacterial pathogens (eg. *Escherichia coli*) [25], this remains unknown for *S. suis*. Nonetheless, bacteria must breach one of the physiological barriers that separate the brain: the blood-CSF barrier is the preferred site of entry into the CNS for most meningitis-causing human bacterial pathogens, which subsequently reach the CSF and the meningeal cells with which they may interact [19]. Bacterial adhesion to the cell surface is considered a critical host-pathogen interaction. Consequently, the adhesion of the *S. suis* strains, belonging to different STs, to human meningeal cells was measured. Since primary human leptomeningeal cells cannot be reliably cultured, previously established human leptomeningioma cell cultures were used [26]. These cells present the same cytological and morphological structures as their primary counterparts [24] and have been used with various meningitis-causing human bacterial pathogens [21, 23]. The ST1, ST7, ST25, and ST28 strains all adhered to meningeal cells, with associated bacteria ranging between 5 × 10^4^ colony forming units (CFU) and 1 × 10^7^ CFU, equivalent to 1 and 2500 bacteria/cell, respectively (Fig. 1A). Though significant differences were not observed between STs, there were differences between strains: P1/7 and 89–1591 adhered significantly less and more than the other strains (p < 0.01), respectively. These differences suggest the partial implication of strain-specific factors [1], which remain largely unknown since nearly all virulence studies have been conducted using ST1, and more recently, ST7 strains [1]. Consequently, very little is known regarding ST25 and ST28-specific virulence factors, including those implicated in adhesion. The adhesion levels of *S. suis* are similar to those reported for group B *Streptococcus* (GBS) serotypes III and V, yet *S. suis* adhered more strongly than other important encapsulated human bacterial pathogens, such as *Streptococcus pneumoniae* serotype 2, *Haemophilus influenzae* type b and *E. coli* K1, but less so than *Neisseria meningitidis* serogroup B, when similar initial concentrations were used [21, 23]. This adhesion capacity of *S. suis* serotype 2 had also been observed when porcine tracheal epithelial cells, human lung epithelial cells, hBMEC or pBMEC were infected with ST1 strains [11, 13, 27, 28]. Importantly, this is the first time that the interactions between *S. suis* serotype 2 ST25 or ST28 strains and CNS cells, other than the BMECs, have been studied, regardless of the species. Unlike with human and porcine intestinal epithelial cells [29], no differences were observed between adhesion of human or porcine *S. suis* serotype 2 strains to human meningeal cells.

Since *S. suis* adhered to meningeal cells, its capacity to subsequently invade the cells was evaluated. Bacterial invasion ranged between 2 × 10^3^ and 7 × 10^3^ CFU for the different strains, equivalent to 0.05 and 0.2 intracellular bacterium/cell, respectively, with the exception of the ST7 strain, for which 8 × 10^4^ CFU were recovered, equivalent to 2 intracellular bacteria/cell (Fig. 1B). Unfortunately, the definition of bacterial invasion remains controversial and subjective, with no criterion allowing to determine with certainty if a bacterial strain is invasive [30]. Though the number of internalized bacteria required for *S. suis* serotype 2 to induce meningitis remains unknown, it was previously suggested that the survival of a single intracellular *H. influenzae* may result in meningitis [31]. Consequently, biologically relevant invasion was defined as 1 intracellular bacterium/cell in this study. As such, only the ST7 was capable, in theory, of meningeal cell invasion: this capacity could be the result of unique factors, since important differences have been observed in the genome of this strain when compared to ST1 and ST25 strains [32]. The general inability of *S. suis* to invade meningeal cells is a characteristic shared with GBS, *S. pneumoniae* and *H. influenzae* [21, 23]. By contrast, *N. meningitidis* and *E. coli* invaded meningeal cells [23]. It was previously demonstrated that though *S. suis* serotype 2 invaded porcine tracheal epithelial cells, it did not invade human lung epithelial cells or hBMEC; invasion of pBMEC remains controversial however [11, 13, 27, 28, 30]. Breaching of the meningeal cell barrier via the paracellular route cannot be discarded however, since this route is difficult to evaluate in the absence of transwell inserts and measuring of the transepithelial electrical resistance. Although described for intestinal epithelial cells [29], this route of entry has not yet been described for *S. suis* in the CNS, where bacteria were reported to use the transcellular route (cellular invasion) [11, 14, 33]. Surprisingly, a correlation between meningeal cell adhesion and invasion by the strains studied was not observed, suggesting that different bacterial and/or host cell factors are probably involved in these interactions.

Of the different well-characterized *S. suis* serotype 2 virulence factors, the CPS has, amongst its various properties, been described to modulate the adhesion to and invasion of epithelial and endothelial cells [27, 29, 30, 34]. Meanwhile, the SLY has more recently been suggested to be involved in the adhesion to and invasion of human epithelial cells at sub-cytolytic concentrations [35]. Finally, the roles of cell wall modifications, including the D-alanylation of the LTA and N-acetylation of the PGN, in adhesion and invasion have been studied using pBMEC and porcine tracheal epithelial cells [8, 28]. Given these properties, the role of these factors on the adhesion to and invasion of meningeal cells by *S. suis* was determined. The adhesion of *S. suis* to meningeal cells was significantly modulated by the CPS (p < 0.001) (Fig. 1C): the absence of the CPS increased adhesion by tenfold. Meanwhile, the SLY and cell wall modifications had no effect. The absence of the CPS also significantly increased invasion of meningeal cells (Fig. 1D), by one hundredfold (p < 0.001), such that the intracellular bacteria/cell ratio was of 10. These modulations by the CPS, though expected since it is known to mask various surface proteins implicated in adhesion and invasion, are similar to those obtained using pBMEC and porcine tracheal epithelial cells [11, 28, 34]. In fact, P1/7 was non-invasive when encapsulated, but highly invasive in the absence of its CPS. Regulation of CPS expression may play an important role in bacterial-host interactions in vivo [1]. On the other hand, the SLY did not modulate the adhesion to and invasion of pBMEC or porcine tracheal epithelial cells by *S. suis*, as was observed in the present study [11, 28]. Meanwhile, the D-alanylation of the LTA modulated the adhesion to and invasion of pBMEC, but not of porcine tracheal epithelial cells [8, 11, 28]. Consequently, different receptors may be implicated in adhesion and invasion according to the cell type and/or species.

### *Streptococcus suis* serotype 2 adheres to and invades human astrocytes and these interactions are modulated by the CPS and, to a certain extent, by the SLY and cell wall modifications

**Fig. 2:**
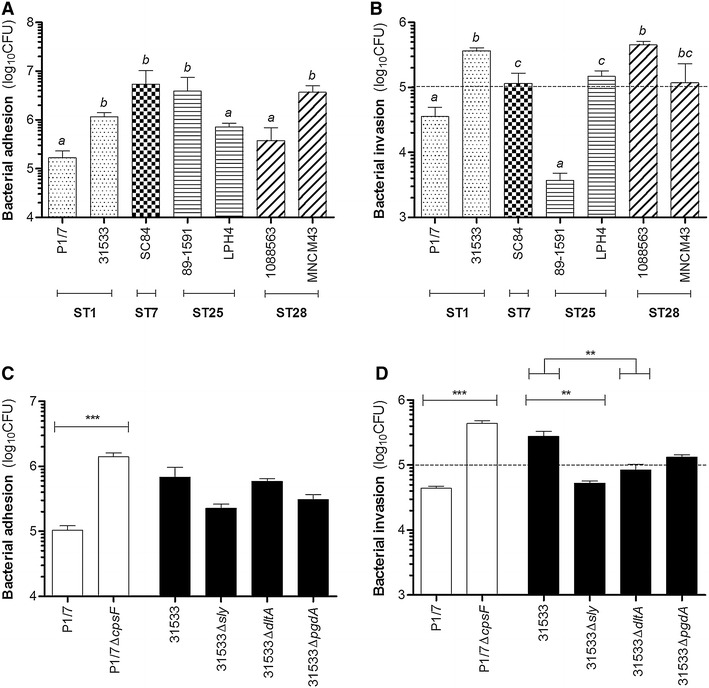
Interactions of *S. suis* serotype 2 with human astrocytes. Bacterial adhesion (**A**) and invasion (**B**) of human astrocytes by the *S. suis* strains belonging to different sequence types (STs), and adhesion (**C**) and invasion (**D**) of human astrocytes by the mutant strains. Results are expressed as log_10_ mean ± SEM CFU obtained from three independent experiments. *Dotted lines* indicate a ratio of 1 intracellular bacterium/cell. The use of different letters (*a*, *b* or *c*) indicates a significant difference between groups (p < 0.01); **(p < 0.01); ***(p < 0.001)

In the case where *S. suis* serotype 2 would modulate its CPS, the pathogen may penetrate the meningeal cells of the pia mater, as observed in this study, in order to reach the astrocytes of the *glia limitans superficialis* [21]. As with meningeal cells, no significant differences were observed between adhesion of the different STs to astrocytes. Indeed, adhesion ranged between 1 × 10^5^ and 5 × 10^6^ CFU, equivalent to 1 and 50 bacteria/cell, respectively (Fig. 2A). Moreover, the strains P1/7, LPH4 and 1088563 adhered similarly, but significantly less than the other strains (p < 0.05). The adhesion levels of *S. suis* serotype 2 to human astrocytes are similar to those previously reported for GBS serotypes III and V [21].

Since all of the *S. suis* strains adhered to astrocytes, their subsequent invasion capacity was evaluated. Unlike with meningeal cells, multiple strains were, in theory, capable of biologically relevant invasion (Fig. 2B). Indeed, at least 1 × 10^5^ CFU, equivalent to 1 intracellular bacterium/cell, were recovered following infection with the different strains, with the exception of P1/7 and 89-1591. Differences were nonetheless observed between the strains capable of invasion: 31533 and 1088563 invaded the cells significantly more than did SC84, LPH4 or MNCM43 (p < 0.01). These differences suggest that invasion of astrocytes by *S. suis* is a strain-specific characteristic: as mentioned previously however, the characteristics of ST25 and ST28 strains remain largely unknown. These results are different from those obtained using murine astrocytes, which did not internalize the *S. suis* serotype 2 strains 31533 (ST1) and SC84 (ST7), also used in this study [4]. On the contrary, invasion of human astrocytes by GBS was reported as infrequent since the intracellular bacterium/cell ratio recovered following infection was much lower than 1 [21]. As with meningeal cells however, *S. suis* adhesion to and invasion of astrocytes did not correlate.

Since *S. suis* interacted with astrocytes, the role of the CPS, SLY and cell wall modifications were evaluated. Adhesion to human astrocytes was significantly modulated by the CPS (p < 0.001) (Fig. 2C), its absence increasing adhesion by tenfold. Meanwhile, the absence of the SLY and cell wall modifications did not modulate the adhesion of *S. suis* to astrocytes. The absence of the CPS also significantly increased the invasion capacity of astrocytes (p < 0.001) (Fig. 2D), for which the intracellular bacteria/cell ratio recovered was of 5, equivalent to a tenfold increase. This greatly differs from murine astrocytes, which *S. suis* did not invade even in the absence of CPS [4]. Unlike with meningeal cells however, the absence of the SLY and D-alanylation of the LTA significantly decreased invasion of astrocytes by *S. suis* (p < 0.01): the strain was no longer capable of biologically relevant cell invasion. Meanwhile, as with meningeal cells, the N-acetylation of the PGN had no effect. These results are in accordance with previous studies: sub-cytolytic levels of SLY increased the invasion capacity of *S. suis* serotype 2 in epithelial cells [35], while, as mentioned above, the D-alanylation of the LTA modulated the invasion of pBMEC [8].

### *Streptococcus suis* serotype 2 induces little inflammatory response from infected human meningeal cells

*Streptococcus suis*-induced inflammation is a characteristic of the infection that can lead to an exacerbated inflammatory response and host death [36]. Furthermore, meningitis, the hallmark of the *S. suis* CNS infection, is defined as an inflammation of the meninges in which the surrounding CNS tissue may be implicated [36]. Consequently, an understanding of the role of these cells in the production of inflammatory mediators following *S. suis* serotype 2 infection is crucial.

**Fig. 3:**
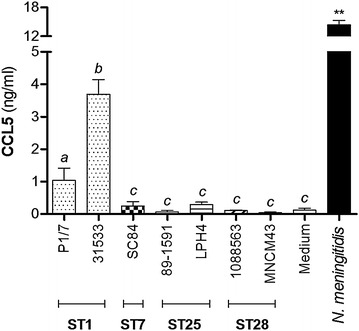
Only *S. suis* serotype 2 ST1 strains induce CCL5 from infected human meningeal cells. CCL5 production from human meningeal cells infected with the *S. suis* strains belonging to different sequence types (STs). Results are expressed as mean ± SEM ng/ml obtained from three independent experiments. The use of different letters (*a*, *b* or *c*) indicates a significant difference between groups (p < 0.01). **Indicates a significant difference between *N. meningitidis* and the medium (p < 0.01)

Human meningeal cells have been previously reported to produce the pro-inflammatory cytokine interleukin (IL)-6 and chemokines C–C motif ligand (CCL)2, CCL5 and C–X–C motif ligand (CXCL)8 in response to meningeal pathogens, but not IL-1α, IL-1β, IL-10, IL-12, tumor necrosis factor (TNF)-α, CCL3 or CCL4 [21–23]. Consequently, only the production of IL-6, CCL2, CCL5, and CXCL8 was quantified 24 h following infection with the different *S. suis* strains. Meningeal cells infected with *N. meningitidis*, used as a positive control, significantly produced all four mediators (p < 0.01). However, no production of IL-6, CCL2 or CXCL8 was observed when cells were infected with *S. suis*, regardless of the strain (data not shown). Interestingly, a significant production of CCL5 was induced, but by both ST1 strains only (p < 0.01) (Fig. 3). The levels were significantly greater when cells were infected with 31533 than with P1/7 (p < 0.01). This production of CCL5 corroborates with previous results in which elevated levels of the mediator were observed in the CSF of human patients with bacterial meningitis [37]. Furthermore, CCL5 is implicated in the chemoattraction of monocytes, which massively infiltrate the SAS and meninges during *S. suis* meningitis [36]. An absence of these four inflammatory mediators was also observed when human meningeal cells were infected with GBS or *S. pneumoniae* [21, 23]. By contrast, these cells produced important levels of the four mediators following infection with *N. meningitidis* and *H. influenzae*, but only produced CCL2 and CXCL8 following infection with *E. coli* [23]. However, *S. suis* serotype 2 induced IL-6, CCL2 and CXCL8 from hBMEC and IL-6 and CXCL8 from pBMEC, suggesting a possible cell and/or species specificity [38, 39].

Given the relative absence of the inflammatory mediators evaluated, the *S. suis*-induced cytotoxicity was evaluated (Table 1). Cytotoxicity levels were below 10 % following infection with the different strains, suggesting that unlike with the hBMEC and pBMEC, the SLY, produced by the ST1 and ST7 strains but not by the ST25 or ST28 strains, was not toxic for human meningeal cells. Consequently, the absence of the meningeal cell inflammatory mediators evaluated was probably not due to cell death. It is possible that human meningeal cells do not express certain of the various receptors implicated in the *S. suis* serotype 2 recognition, including TLRs (TLR2, TLR4, TLR9, and possibly, TLR3) and NLRs (NOD2) [40]. Of these however, the TLR2, often considered as the most important to the *S. suis* serotype 2 pathogenesis, and TLR4, are present on the cell surface [1, 41].

**Table 1:** *Streptococcus suis* serotype 2-induced human meningeal cell and astrocyte cytotoxicity, 24 h following infection

Strain	Sequence type	Presence of SLY	% cytotoxicity ± SEM (n = 3)	
			Meningeal cells	Astrocytes
P1/7	1	Yes	0.1 ± 0.1	38.2 ± 3.4
31533	1	Yes	3.4 ± 1.2	63.3 ± 0.6
SC84	7	Yes	9.1 ± 2.6	35.3 ± 6.2
89-1591	25	No	0.0 ± 0.0	71.5 ± 1.5
LPH4	25	No	6.1 ± 2.2	28.9 ± 6.0
1088563	28	No	0.0 ± 0.0	12.0 ± 3.9
MNCM43	28	No	0.0 ± 0.0	1.9 ± 1.0
*N. meningitidis*	Not applicable	Not applicable	Not determined	88.6 ± 0.9

### *Streptococcus suis* serotype 2 does not induce an inflammatory response from infected human astrocytes

Human astrocytes produce various inflammatory mediators following bacterial infection, including IL-6, CCL2, CCL5, and CXCL8 [21]. Consequently, these mediators were quantified following infection with the different *S. suis* strains. As with meningeal cells, *N. meningitidis* induced a significant production of all four mediators (p < 0.01). Meanwhile, no production of IL-6, CCL2, CCL5 or CXCL8 was observed when cells were infected with *S. suis*, regardless of the strain (data not shown). Interestingly, these results vary from those obtained using murine astrocytes, which were an important source of TNF-α, IL-6, CCL2, and CXCL1 following *S. suis* serotype 2 infection [4]. Furthermore, recognition of *S. suis* serotype 2 by murine astrocytes was mainly TLR2-dependent, since the expression of TLR1, TLR4 and TLR6 was barely modulated following infection [4]. Meanwhile *S. suis* serotype 2-induced TNF-α and CCL2 were partially dependent on TLR2 in murine astrocytes [4]. By contrast, in the present study, no CCL2 production was observed when human astrocytes were infected with *S. suis* serotype 2, while production of TNF-α was not determined. Consequently, the species from which the cells originate appears to play an important role on the results obtained, as the receptors present may vary, which could influence the subsequent inflammatory response [42, 43].

Given the absence of the inflammatory mediators measured, *S. suis*-induced astrocyte cytotoxicity was also evaluated (Table 1). In contrast to the lack of meningeal cell death following infection with *S. suis*, astrocytes were generally more sensitive to infection. The ST1, ST7 and ST25 strains induced important cytotoxicity, which ranged between approximately 30 and 70 %, while the ST28 strains caused little cytotoxicity (less than 15 %). The SLY may be partially responsible for the *S. suis*-induced astrocyte cell death since the ST1 and ST7 strains induced higher cytotoxicity levels than did the SLY-negative strain LPH4. Furthermore, the differences observed between the ST1 and ST25 strains suggest that strain-specific factors are implicated in these interactions. Nevertheless, the *S. suis*-induced astrocyte cell death was probably not responsible for the absence of the inflammatory mediators evaluated, since *N. meningitis* caused near total cell death, with 90 % cytotoxicity, yet induced important levels of these inflammatory mediators.

## Conclusions

*Streptococcus suis* serotype 2 interacted with human meningeal cells and astrocytes via the adhesion to both cell types; invasion of meningeal cells was rare however, whereas that of astrocytes was generally more frequent. Furthermore, these interactions were largely modulated by the CPS, and to a certain extent, by the SLY and cell wall modifications. Regardless of the fact that *S. suis* interacted with meningeal cells and astrocytes, little or no production of the inflammatory mediators evaluated was observed. This suggests that the meningeal cells of the leptomeninges and the astrocytes of the underlying *glia limitans superficialis* may not be directly implicated in the inflammatory response observed during *S. suis* meningitis in humans. Nevertheless, the use of a microarray or proteomic assay investigating a larger number and greater variety of inflammatory mediators induced by *S. suis* serotype 2 in these cell types may yield further details. Alongside, a given ST could not be correlated with a specific interaction since important variations were observed between strains within a single ST. Consequently, the interactions with meningeal cells and astrocytes, though important for the pathogenesis, are not ST-dependent but rather a characteristic of *S. suis* serotype 2 that varies according to the strain.

## Methods

### Bacterial strains and growth conditions

The well-encapsulated *S. suis* serotype 2 strains and isogenic mutants for different well-characterized virulence factors used in this study are listed in Table 2. Bacteria were grown overnight on Columbia Blood Agar containing 5 % sheep blood (v/v) at 37 °C with 5 % CO_2_ (Oxoid, Basingstoke, UK). Five ml of Todd-Hewitt Broth (THB; Becton–Dickinson, Swindon, UK) were inoculated and incubated for 8 h at 37 °C with 5 % CO_2_. Working cultures were prepared by inoculating 30 ml of THB with 10 µl of a 10^−3^ dilution of the 8 h cultures, incubated for 16 h at 37 °C with 5 % CO_2_. Bacteria were washed twice with pH 7.3 phosphate-buffered saline (PBS), resuspended in cell culture medium, appropriately diluted, and plated on Todd-Hewitt Broth agar (THA) to accurately determine concentrations. The *N. meningitidis* serogroup B strain MC58 [44], used as a positive control, was grown on supplemented GC agar and bacterial suspensions prepared in cell culture medium, as previously described [24].

**Table 2:** *Streptococcus suis* serotype 2 strains used in this study

Strain	Sequence type	Country	Host	Phenotype	References
P1/7	1	United Kingdom	Pig	Wild-type	[46]
P1/7Δ*cpsF*	1	–	–	Non-encapsulated mutant	[6]
31533	1	France	Pig	Wild-type	[47]
31533Δ*sly*	1	–	–	Suilysin-deficient mutant	[7]
31533Δ*dltA*	1	–	–	D-alanylation of lipoteichoic acid-deficient mutant	[8]
31533Δ*pgdA*	1	–	–	N-deacetylation of peptidoglycan-deficient mutant	[9]
SC84	7	China	Human	Wild-type	[48]
89-1591	25	Canada	Pig	Wild-type	[49]
LPH4	25	Thailand	Human	Wild-type	[50]
1088563	28	Canada	Pig	Wild-type	[51]
MNCM43	28	Thailand	Human	Wild-type	[50]

### Human meningeal cell and astrocyte cultures

Human meningioma cells, obtained from surgically removed tumors as previously described, express the characteristic markers of desmosomal desmoplakin, epithelial membrane antigen, vimentin, and cytokeratin [24]. The cells were grown in Dulbecco’s Modified Eagle Medium containing Glutamax-1 and sodium pyruvate (Lonza, Slough, UK) supplemented with 10 % fetal calf serum (v/v) (FCS; Lonza) and seeded in flasks pre-coated with 5 mg/cm^2^ of type I collagen from rat tail (Becton–Dickinson); culture passages 2–10 were used [24]. SVGmm human fetal astrocyte cells were grown in Eagle’s Minimal Essential Medium (Lonza) supplemented with 10 % FCS (v/v) [45].

### Infection of human meningeal cells and astrocytes

Human meningeal cells and astrocytes were grown to confluence in 24-well cell culture plates, averaging 4 × 10^4^ cells/well and 1 × 10^5^ cells/well, respectively. Cell monolayers were maintained overnight in medium containing 1 % FCS (v/v) and washed twice with warm PBS prior to bacterial challenge, in triplicate, with the different *S. suis* serotype 2 wild-type or mutant strains, or *N. meningitidis*. Based on preliminary assays, 1 ml of 4 × 10^4^ CFU/well for meningeal cells and 1 × 10^5^ CFU/well for astrocytes [multiplicity of infection (MOI) = 1] was added for bacterial adhesion. For bacterial invasion, host cell cytotoxicity and inflammatory activation, 4 × 10^6^ CFU/well for meningeal cells and 1 × 10^7^ CFU/well for astrocytes (MOI = 100) were added. Monolayers were incubated for 9 h (adhesion and invasion) or 24 h (cell cytotoxicity and inflammatory activation) at 37 °C with 5 % CO_2_ as previously described [21–23].

### Bacterial adhesion and invasion measurement

After incubation, cells were washed four times with warm PBS, lysed using 250 µl/well of 1 % saponin (w/v) (Sigma-Aldrich, Dorset, UK) in PBS and incubated for 15 min at 37 °C with 5 % CO_2_. Bacterial adhesion was measured by plating the lysates onto THA and incubating overnight at 37 °C with 5 % CO_2_. Bacterial invasion was carried out using the antibiotic protection assay [5]: cell medium was removed after 9 h, monolayers were washed twice with warm PBS and 1 ml of medium containing 5 µg/ml penicillin G (Sigma-Aldrich) and 100 µg/ml of gentamicin (Sigma-Aldrich) was added for 90 min at 37 °C with 5 % CO_2_ to kill extracellular bacteria. The last wash was plated to confirm antibiotic activity.

### Host cell cytotoxicity assay

Lactate dehydrogenase (LDH) release was measured 24 h post-infection using the CytoTox 96 Non-Radioactive Cytotoxicity Assay kit (Promega, UK) according to instructions. Non-infected monolayers were included for measurement of spontaneous LDH release and maximum LDH release was induced using the lysis reagent included in the kit. The absorbance was read at 492 nm using the iMark Absorbance Reader (Bio-Rad, Hercules, CA, USA).

### Host cell inflammatory activation

The measurement of the pro-inflammatory cytokine IL-6 and chemokines CCL2, CCL5 and CXCL8 was carried out by enzyme-linked immunosorbent assay as previously described [22].

### Statistical analyses

Unpaired t-tests, Mann–Whitney rank sum tests and one-way ANOVA, where appropriate, were performed to find statistical differences between groups. p < 0.05 was considered statistically significant.

## Abbreviations

CCL: C–C motif ligand
CFU: colony forming unit
CNS: central nervous system
CPEC: choroid plexus epithelial cells
CPS: capsular polysaccharide
CSF: cerebrospinal fluid
CXCL: C–X–C motif ligand
FCS: fetal calf serum
GBS: group B *Streptococcus*
hBMEC: human brain microvascular endothelial cells
IL: interleukin
LDH: lactate dehydrogenase
LTA: lipoteichoic acid
MOI: multiplicity of infection
NOD: nucleotide-binding oligomerization domain
NLR: NOD-like receptors
pBMEC: porcine brain microvascular endothelial cells
PBS: phosphate-buffered saline
PGN: peptidoglycan
SAS: subarachnoid space
SLY: suilysin
ST: sequence type
THA: Todd-Hewitt Broth agar
THB: Todd-Hewitt Broth
TLR: toll-like receptor
TNF: tumor necrosis factor
v/v: volume/volume
w/v: weight/volume
